# The effects of growth and fixed mindsets on academic procrastination among sports science students: the mediating role of locus of control

**DOI:** 10.3389/fpsyg.2026.1932410

**Published:** 2026-10-29

**Authors:** Burak Öner, Aykut Şahin

**Affiliations:** 1 Department of Property Protection and Security, Çemişgezek Vocational School, Munzur University, Tunceli, Türkiye; 2 Department of Physical Education and Sport, Faculty of Sports Sciences, Munzur University, Tunceli, Türkiye

**Keywords:** academic procrastination, achievement beliefs, educational outcomes, higher education, implicit theories of intelligence, locus of control, parallel mediation

## Abstract

**Purpose:**

Academic procrastination (AP) is a pervasive self-regulatory problem that undermines students’ academic functioning and psychological well-being. This study examined the direct and indirect relationships between growth and fixed mindsets, internal and external locus of control, and AP in sports science students.

**Method:**

Internal and external locus of control were tested as parallel mediators in separate growth and fixed mindset models. A cross-sectional predictive design was employed with 544 undergraduate sports science students in Eastern Anatolia, Türkiye. Participants completed measures of mindset, locus of control, and AP. Parallel mediation analyses were conducted using PROCESS Macro (Model 4) with 5,000 bootstrap samples, controlling for age and gender.

**Results:**

The direct effects indicated that a growth mindset was negatively associated with AP, whereas a fixed mindset was positively associated with AP. A growth mindset was positively associated with an internal locus of control and negatively associated with an external locus of control, whereas a fixed mindset was negatively associated with an internal locus of control and positively associated with an external locus of control. An external locus of control was positively associated with AP and significantly mediated the relationships between both mindset dimensions and AP. In contrast, the internal locus of control did not significantly mediate either relationship. Male students reported higher AP levels than female students, and age was negatively associated with AP.

**Conclusion:**

These findings suggest that an external locus of control is a key behavioral mechanism linking mindset beliefs to AP. Interventions targeting maladaptive external control beliefs may help reduce AP among sports science students.

## 1 Introduction

Academic procrastination (AP) is defined as the voluntary postponement of academic tasks by students, even though it is predicted that the delay in acting on an intended study will lead to worse outcomes (Steel, 2007). AP is common among university students, and its rate has increased over time (Beutel et al., 2016; Fernie et al., 2017; Johansson et al., 2023; Steel, 2007). When procrastination behaviors become more persistent among students, they can lead to academic failure (Bahl et al., 2025; Fernie et al., 2017; Kim and Seo, 2015; Steel, 2007), problems related to quality of life, and negative health outcomes (Johansson et al., 2023; Ren et al., 2021; Stead et al., 2010). Therefore, it is important to reduce AP behaviors to support the well-being, wellness, and life satisfaction of university students and enhance their academic performance.

Steel (2007) emphasized that procrastination may stem from mental processes, including irrational beliefs, and focused on how an individual’s cognitive functioning shapes procrastination behavior. Moreover, Steel (2007) proposed that students’ AP behaviors cannot be attributed solely to time-management issues, but that self-regulation deficits and impulsivity can also lead to procrastination. Mindset Theory (Dweck, 2006) is one of the frameworks that can contribute to our understanding of university students’ AP behaviors by focusing on their mindset-based beliefs. According to the theory, a growth mindset represents the belief that qualities and abilities can be developed through effort, determination, and appropriate strategies, whereas a fixed mindset represents the belief that qualities such as intelligence, personality, and character are innate, fixed, and immutable. Unlike students with a fixed mindset, those with a growth mindset view failure as a natural part of the learning process and perceive challenges as opportunities for development (Yeager and Dweck, 2012). According to Dweck (2006), these two mindsets deeply affect not only one’s learning process but every aspect of life—from school to sports and from the business world to relationships. Students with a growth mindset demonstrate productive behaviors, such as greater resilience to challenges, more active engagement in academic tasks, and a lower tendency toward AP (Muenks et al., 2024; Yeager and Dweck, 2012). In contrast, students with a fixed mindset tend to attribute failure to external factors rather than to a lack of effort or determination, resulting in negative cognitive and affective responses during academic tasks. This may increase students’ tendency to display more AP behaviors in the form of avoidance of challenging academic tasks (Burnette et al., 2013; Yeager and Dweck, 2012). For example, a study conducted among university students confirmed that as belief in a growth mindset and the developmental nature of challenge increased, AP decreased, whereas as a fixed mindset and perceiving challenge as a threat increased, procrastination increased (Muenks et al., 2024).

Psychological factors are also among the main influences on university students’ postponement of fulfilling an obligation or developing an action (Araya-Castillo et al., 2023). From a psychological perspective, one of the motivational dimensions related to AP is locus of control (LoC) (Steel and Klingsieck, 2016). Although mental beliefs are linked to outcomes associated with procrastination, the psychological mechanisms through which these beliefs are associated with procrastinatory behavior have not been sufficiently understood. Understanding the mechanisms through which mindset beliefs are associated with procrastination behavior may provide a more comprehensive explanation of why some students postpone academic tasks more than others. A potential mechanism is LoC, which reflects individuals’ beliefs about the extent to which outcomes are determined by their actions or by external factors. According to the Social Learning Theory, LoC encompasses generalized expectations regarding whether the outcomes one achieves are a result of one’s own behavior (internal) or of chance, fate, or powerful external forces (external) (Rotter, 1966, 1975).

It has been proposed that individuals’ beliefs about intelligence precede and shape the development of their LoC (Dweck et al., 1995). According to this theoretical framework, individuals who believe that intelligence is fixed are expected to develop an external LoC by attributing success or failure to factors beyond their control, whereas individuals who believe that intelligence is malleable are expected to develop an internal LoC by believing that effort determines outcomes (Bodill and Roberts, 2013). Rotter (1966) notes that believing in luck or fate (external LoC) may function as a means to “curtail sustained endeavor” in some individuals. This describes the motivational state of those who delay the initiation or continuation of a task. Therefore, it can be said that individuals with a high internal LoC tend to assume greater responsibility and exert more effort. Previous studies have reported that university students who form a direct link between their own behavior and outcomes (internal locus) are much less prone to procrastination. In contrast, it has been demonstrated that students who attribute outcomes to luck, fate, or others’ control (external locus) procrastinate at every stage of academic tasks to a greater extent (Bahl et al., 2025; Carden et al., 2004; Certel et al., 2017; Janssen and Carton, 1999). Taken together, the theoretical link between mindset beliefs and LoC and the documented association between LoC and AP provide a rationale for examining LoC as a potential intervening pathway linking mindset orientations to AP, rather than merely as another correlate of procrastination.

Previous research has documented associations between mindset orientations and AP (Lou and Li, 2023; Macnamara and Burgoyne, 2023) and between LoC and AP (Certel et al., 2017; Mataracı and Alemdağ, 2024). However, these relationships have largely been examined separately, leaving unclear whether internal and external LoC represent distinct pathways linking beliefs about the malleability of abilities with AP. Examining both forms of LoC simultaneously as parallel mediators may therefore provide a more differentiated account of the indirect associations between mindset orientations and AP.

Accordingly, the present study examined the indirect associations between growth and fixed mindsets and AP through internal and external LoC among sports science students. Focusing on sports science students also provides an opportunity to examine the proposed psychological framework within a specific educational context. Students’ beliefs about the development of their abilities and their perceptions of personal control may be particularly relevant to how they approach academic demands and regulate their academic behavior. Examining the relationships among mindset, LoC, and AP in this population therefore extends the existing literature by considering the proposed psychological mechanism in a less frequently examined group, while not assuming that these relationships are unique to sports science students. By integrating mindset theory with perceived control, the present study provides a theoretically grounded framework for examining how mindset orientations may be associated with AP through distinct forms of LoC. Distinguishing between internal and external LoC allows these pathways to be examined separately and may provide a more differentiated understanding of the relationship between mindset orientations and AP.

The hypothesized structural relationships and parallel mediation mechanisms for both mindset models are shown in Figures 1 and 2, respectively. Accordingly, the following specific hypotheses were developed to empirically test each model among sports science students:

**Figure 1:**
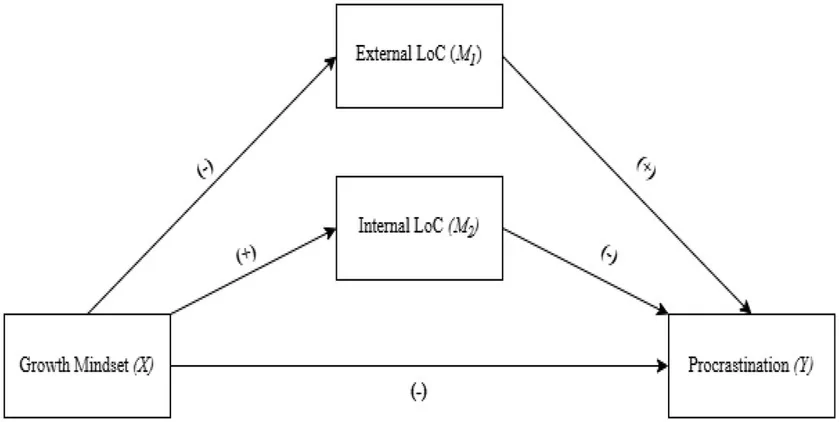
Hypothesized Parallel Mediation Model of Growth Mindset, LoC, and AP (Model 1).

**Figure 2:**
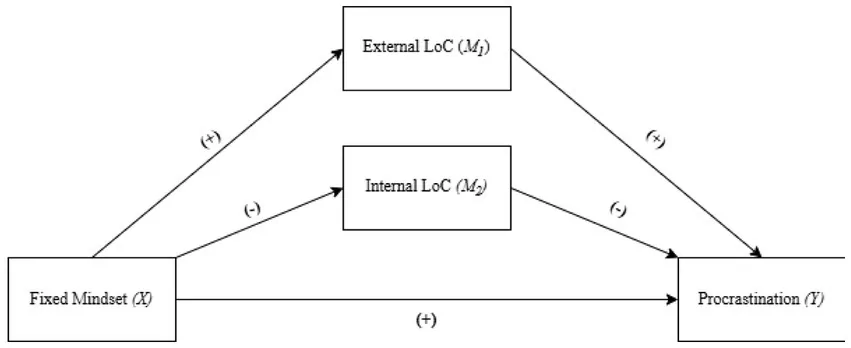
Hypothesized Parallel Mediation Model of Fixed Mindset, LoC, and AP (Model 2).

*H1*: A growth mindset is negatively associated with AP among sports science students.

*H2*: Internal LoC mediates the relationship between growth mindset and AP among sports science students.

*H3*: External LoC mediates the relationship between growth mindset and AP among sports science students.

*H4*: A fixed mindset is positively associated with AP among sports science students.

*H5*: Internal LoC mediates the relationship between a fixed mindset and AP among sports science students.

*H6*: External LoC mediates the relationship between a fixed mindset and AP among sports science students.

## 2 Materials and methods

### 2.1 Study design and participants

In this study, a cross-sectional predictive design was used to examine the mediating role of academic LoC in the relationship between mindset and AP among sports science students. A convenience sampling method was employed to select participants from among the sports science students. A total of 544 sports science students from Munzur and Fırat universities in the Eastern Anatolia Region of Turkey participated in the study. An *a priori* power analysis was not conducted before data collection. However, the final sample size (N = 544) was considered adequate in light of simulation-based research on mediation analysis, which shows that required sample sizes vary according to the magnitude of the indirect-effect paths and model complexity (Fritz and MacKinnon, 2007; Schoemann et al., 2017). The present sample was substantially larger than the sample sizes reported for detecting indirect effects of small-to-moderate magnitude using bootstrap-based mediation analyses.

The participants’ ages ranged from 18 to 45 years (Mage = 21.98, SD = 3.88). Of the students, 56.8% (*n* = 309) were male, and 43.2% (*n* = 235) were female. The sample consisted of 167 first-year students (30.7%), 64 s-year students (11.8%), 173 third-year students (31.8%), and 140 fourth-year students (25.7%). Regarding the department of study, 87 participants (16.0%) were enrolled in Physical Education Teaching, 180 (33.1%) in Sports Management, 207 (38.1%) in Coaching Education, and 70 (12.9%) in Recreation.

### 2.2 Measures

#### 2.2.1 Growth and fixed mindset

The 13-item Turkish Mindset Theory Scale (MTS) was used to measure participants’ Growth and Fixed mindset structures. The scale has a four-dimensional structure consisting of Procrastination (four items), Immutability of Belief (three items), Belief in Improvement (three items), and Effort (three items). The Belief in Improvement and Effort subdimensions represent the growth mindset, while the Procrastination and Immutability of Belief subdimensions represent the fixed mindset. Accordingly, the Growth Mindset score was calculated by averaging the items of the Belief in Improvement and Effort subdimensions, whereas the Fixed Mindset score was calculated by averaging the items of the Procrastination and Immutability of Belief subdimensions. Each mindset item is rated on a 5-point Likert scale ranging from 1 (“Not suitable for my thoughts at all”) to 5 (“It is very suitable for my thoughts”). Thus, higher mean scores indicate that the corresponding mindset structure is more dominant in the participant (Yilmaz, 2022). Second-order confirmatory factor analysis (CFA) supported the two higher-order structures of Growth Mindset and Fixed Mindset, with acceptable fit indices (Table 1). In this study, Cronbach’s α coefficient was 0.83 for growth mindset and 0.84 for fixed mindset.

**Table 1:** Fit indices of the MTS, AloCS, and APS (*n* = 544).

Fit indices	MTS values	ALoCS values	APS values	Acceptable values
χ2 / df	3.64	3.67	3.82	<5
RMSEA	0.07	0.07	0.07	<0.08 good fit <0.10 poor fit
SRMR	0.05	0.04	0.02	<0.08
CFI	0.94	0.93	0.99	≥0.90
GFI	0.93	0.91	0.98	≥0.90
TLI	0.92	0.91	0.97	≥0.90
NFI	0.92	0.90	0.98	≥0.90

MTS, Mindset Theory Scale; ALoCS, Academic Locus of Control Scale; APS, Academic Procrastination Scale; χ^2^/df: Chi-square/degrees of freedom; RMSEA, Root mean square error of approximation; SRMR, Standardized root mean square residual; CFI, Comparative fit index; GFI, Goodness-of-fit index; TLI, Tucker-Lewis index, NFI, Normed fit index.

#### 2.2.2 Internal-external locus of control

The 17-item Turkish Academic Locus of Control Scale (ALoCS) was used to measure the participants’ internal and external control beliefs regarding academia. The scale has a two-dimensional structure: internal LoC (six items) and external LoC (eleven items). Each LoC item was rated on a 5-point Likert scale ranging from 1 (“Not at all appropriate”) to 5 (“Completely appropriate”). Evaluations of internal and external LoC beliefs are based on the total score, with higher scores indicating that the relevant LoC beliefs are more predominant in the participant (Akın, 2007). In this study, Cronbach’s α coefficient was 0.88 for internal LoC and 0.90 for external LoC. Additionally, the CFA findings supported the scale’s construct validity (Table 1).

#### 2.2.3 Academic procrastination

The 5-item Academic Procrastination Scale (APS) short form was used to measure participants’ AP behavior. The original form of the scale, validated by Yockey (2016), was adapted into Turkish by Balkis and Duru (2021). Each item on the scale, which represents a unidimensional structure, is assessed using a 5-point Likert scale ranging from 1 (“strongly disagree”) to 5 (“strongly agree”). The assessment of AP behavior is based on the total score, with higher scores indicating an increase in procrastination behavior among participants. In this study, Cronbach’s α coefficient for AP was 0.87, indicating good reliability. The CFA showed that the fit indices for the scale were within acceptable limits (Table 1).

### 2.3 Data collection and procedure

Data were collected using paper-and-pencil surveys administered face-to-face during the 2025–2026 academic year. Prior to data collection, the participants were informed about the purpose of the study, that participation was voluntary, and that their responses would be kept confidential. Only students who voluntarily agreed to participate were included in the study.

The questionnaires were administered in a classroom environment under the supervision of the researchers. Participants completed the questionnaire individually, and no personally identifiable information was collected to ensure anonymity. The questionnaire took approximately 10–15 min to complete. The completed forms were collected on-site by the researchers. To reduce the potential for common method bias, several procedural precautions were implemented during data collection. Participation was anonymous, clear instructions were provided, and questionnaire items were presented in a concise and comprehensible manner. In addition, the specific relationships examined in the study were not disclosed to participants. These procedures were implemented to reduce potential response-related sources of common method bias (Kock et al., 2021).

A total of 586 questionnaires were distributed. During initial data screening, 18 questionnaires were excluded due to missing responses. An additional 24 questionnaires were excluded because respondents had selected the same response option across all questionnaire items, as identified through manual inspection. These cases were treated as uniform response patterns. No predefined threshold or formal statistical procedure was used to identify these patterns. Accordingly, 42 questionnaires were excluded, resulting in a final analytical sample of 544 participants.

Ethical approval for the study was obtained from the relevant institutional ethics committee prior to the data collection. All procedures were conducted in accordance with the ethical principles of the Declaration of Helsinki.

### 2.4 Data analysis

All descriptive and preliminary analyses were performed using IBM SPSS Statistics version 25.0 software. Within the scope of basic descriptive statistics, means, standard deviations, kurtosis and skewness values, and Pearson’s correlation coefficients were calculated. Multicollinearity was assessed using tolerance and variance inflation factor (VIF) values. Confirmatory factor analyses (CFA) were conducted separately for each measurement instrument using IBM SPSS AMOS with maximum likelihood (ML) estimation to examine construct validity. Model fit was evaluated using the χ^2^/df, CFI, TLI, GFI, NFI, RMSEA, and SRMR indices. The criteria for acceptable model fit were χ^2^/df < 5, CFI, TLI, GFI, and NFI ≥ 0.90, and RMSEA and SRMR <0.08 (Hooper et al., 2008; Hu and Bentler, 1999). Composite reliability (CR), average variance extracted (AVE), and Cronbach’s alpha (α) coefficients were calculated to assess reliability and validity. CR values ≥0.70 were considered indicative of adequate composite reliability, whereas AVE ≥ 0.50 was used as the conventional criterion for convergent validity. When AVE was below 0.50, it was interpreted in conjunction with CR rather than considered sufficient on its own to establish convergent validity (Fornell and Larcker, 1981). Parallel mediation analyses to test the proposed models were conducted using Model 4 in the PROCESS Macro 4.2 add-on for SPSS (Hayes, 2022). Standardized regression coefficients ( β ) are reported for individual regression paths, whereas unstandardized coefficients ( B ) obtained from the PROCESS output are reported for total, direct, and indirect mediation effect estimates. The hypothesized mediation models were analyzed using 5,000 bootstrap resamples with percentile-based 95% confidence intervals (CIs). The significance of the indirect effects was assessed based on whether the 95% bootstrap CIs included zero (Hayes, 2022). Gender and age were included as covariates in all mediation analyses to account for their potential associations with AP. Two separate parallel mediation models were specified. In the first model, growth mindset was the predictor, internal and external LoC were entered simultaneously as parallel mediators, and AP was the outcome. In the second model, fixed mindset replaced growth mindset as the predictor, while the mediators and outcome remained the same. Age and gender were included as covariates in both models to account for their potential associations with the mediators and AP. Gender was dummy-coded as 1 for male and 0 for female.

## 3 Results

### 3.1 Common method bias (CMB) test

Harman’s single-factor test was conducted using an unrotated principal component analysis of all measurement items. the first component accounted for 26.43% of the total variance, which was below the commonly used 50% threshold for substantial CMB (Kock et al., 2021). Thus, no single factor accounted for the majority of the variance.

### 3.2 Preliminary analyses and correlations

Before proceeding to the main analyses, the assumption of multicollinearity among the predictor variables was tested. The diagnosis of multicollinearity revealed that the tolerance values ranged from 0.705 to 0.870, which is well above the critical threshold of 0.10. Simultaneously, the VIF values ranged from 1.149 to 1.419, which are well below the standard threshold of 5.0. These results indicate that multicollinearity does not threaten the regression models. Table 2 presents the descriptive statistics of the study variables, the findings on construct validity, and the inter-variable correlation matrix. The skewness values for the study variables ranged between −0.85 and 0.73, and the kurtosis values ranged between 1.03 and −0.61. These values indicate that the variables did not deviate significantly from the normal distribution (George and Mallery, 2016). Additionally, the CR values ranged from 0.87 to 0.90, while the AVE values ranged from 0.46 to 0.58. The only AVE value below 0.50 was observed for External LoC (AVE = 0.46), which was accompanied by a high CR value (CR = 0.90). Accordingly, the AVE and CR values were considered together when evaluating the convergent validity of this construct (Fornell and Larcker, 1981). Analyses of correlations among variables revealed that growth mindset was positively associated with internal LoC (*r* = 0.27, *p* < 0.01), negatively associated with external LoC (*r* = −0.21, *p* < 0.01), and negatively associated with AP (*r* = −0.12, *p* < 0.01). Additionally, a fixed mindset showed significant negative associations with internal LoC (*r* = −0.24, *p* < 0.01), positive associations with external LoC (*r* = 0.36, *p* < 0.01), and positive associations with AP (*r* = 0.28, *p* < 0.01). Furthermore, internal LoC was negatively associated with AP (*r* = −0.15, *p* < 0.01), whereas external LoC was positively associated with AP (*r* = 0.41, *p* < 0.01).

**Table 2:** Correlation analysis, descriptive statistics, and construct validity (*n* = 544).

Variable	1	2	3	4	5
Growth Mindset	—				
Fixed Mindset	−0.297^**^	—			
Internal LoC	0.269^**^	−0.244^**^	—		
External LoC	−0.210^**^	0.357^**^	−0.484^**^	—	
Procrastination	−0.123^**^	0.277^**^	−0.151^**^	0.412^**^	—
Range	1-5	1-5	1–5	1–5	1–5
Mean	3.96	2.39	3.81	2.30	2.61
SD	0.74	0.75	0.84	0.76	0.94
Skewness	−0.85	0.51	−0.77	0.73	0.48
Kurtosis	1.03	−0.06	0.05	0.26	−0.61
CR	0.87	0.88	0.88	0.90	0.87
AVE	0.53	0.53	0.54	0.46	0.58

**p < 0.01 SD, Standard Deviation; CR, Composite Reliability; AVE, Average Variance Extracted.

### 3.3 Regression and mediation analysis

The results of the regression analysis examining the predictive roles of growth and fixed mindsets on internal LoC, external LoC, and AP, controlling for gender and age, are presented in Table 3.

**Table 3:** Regression results for the growth and fixed mindsets mediation model.

Outcome variables	Predictor Variables	Model	Growth mindset model			Model	Fixed mindset model		
			*β*	*t*	*p*		*β*	*t*	*p*
External LoC (M1)		*R*^2^ = 0.064, *F* = 12.26			<0.001	*R*^2^ = 0.144, *F* = 24.71			<0.001
	Mindset (X)		−0.21	−4.47	<0.001		0.36	7.26	<0.001
	Gender		0.08	1.88	0.061		0.07	1.75	0.081
	Age		−0.11	−3.20	0.001		−0.11	−3.17	0.002
Internal LoC (M2)		*R^2^ = 0.074, F = 10.71*			<0.001	*R^2^ = 0.062, F = 9.13*			<0.001
	Mindset (X)		0.27	5.50	<0.001		−0.24	−4.95	<0.001
	Gender		0.03	0.76	0.445		0.05	1.11	0.270
	Age		0.02	0.68	0.499		0.02	0.55	0.582
Procrastination (Y)		*R^2^ = 0.214, F = 45.24*			<0.001	*R^2^ = 0.233, F = 49.71*			<0.001
	Mindset (X)		−0.06	−1.35	0.179		0.16	3.39	0.001
	Internal LoC		0.05	1.19	0.234		0.06	1.29	0.199
	External LoC		0.40	8.77	<0.001		0.36	7.38	<0.001
	Gender		0.16	4.19	<0.001		0.17	4.26	<0.001
	Age		−0.11	−3.57	<0.001		−0.11	−3.68	<0.001

*β*, standardized regression coefficient; LoC, locus of control; M1, external LoC; M2, internal LoC; X, predictor; Y, outcome. Gender was coded as 1, male and 0, female.

For the Growth Mindset model, external LoC, growth mindset (*β* = −0.21, *t* = −4.47, *p* < 0.001), gender (*β* = 0.08, *p* = 0.061), and age (*β* = −0.11, *p* = 0.001) were significant predictors, and the model explained 6.4% of the variance (*R*^2^ = 0.064, *F* = 12.26, *p* < 0.001). In contrast, internal LoC was positively predicted by a growth mindset (*β* = 0.27, *t* = 5.50, *p* < 0.001), while gender and age were not significant predictors (*R*^2^ = 0.074, *F* = 10.71, *p* < 0.001). Regarding AP, external LoC emerged as a strong positive predictor (*β* = 0.40, *t* = 8.77, *p* < 0.001), whereas internal LoC was not significant (*β* = 0.05, *p* = 0.234). A growth mindset did not directly predict procrastination (*β* = −0.06, *p* = 0.179). Gender was significantly positively associated with AP (*β* = 0.16, *p* < 0.001), and age was significantly negatively associated with AP (*β* = −0.11, *p* < 0.001). The overall model explained 21.4% of the variance in AP (*R*^2^ = 0.214, *F* = 45.24, *p* < 0.001).

For the fixed mindset model, external LoC was positively predicted by a fixed mindset (*β* = 0.36, *t* = 7.26, *p* < 0.001), whereas internal LoC was negatively predicted (*β* = −0.24, *t* = −4.95, *p* < 0.001). For internal LoC, the associations of gender and age were not statistically significant, whereas age was significantly negatively associated with external LoC (*β* = −0.11, *p* = 0.002). The model explained 14.4% of the variance in external LoC (*R*^2^ = 0.144, *F* = 24.71, *p* < 0.001) and 6.2% of the variance in internal LoC (*R*^2^ = 0.062, *F* = 9.13, *p* < 0.001). For AP, external LoC was a significant positive predictor (*β* = 0.36, *t* = 7.38, *p* < 0.001), whereas internal LoC was not (*β* = 0.06, *p* = 0.199). A fixed mindset was significantly and positively associated with procrastination (*β* = 0.16, *t* = 3.39, *p* = 0.001). Gender was significantly positively associated with AP (*β* = 0.17, *p* < 0.001), and age was significantly negatively associated with AP (*β* = −0.11, *p* < 0.001). The model explained 23.3% of the variance in AP (*R*^2^ = 0.233, *F* = 49.71, *p* < 0.001).

Mediation analysis showed that growth mindset had a significant negative total effect on AP (B = −0.170, SE = 0.059, 95% CI [−0.288, −0.053]). However, the direct effect of the growth mindset on AP became non-significant after the inclusion of mediators (*B* = −0.080, SE = 0.059, 95% CI [−0.197, 0.037]) (Table 4). Regarding indirect effects, the total indirect effect of the growth mindset on AP through internal and external LoC was significant (*B* = −0.090, SE = 0.027, 95% CI [−0.147, −0.037]). Specifically, external LoC emerged as a significant mediator (*B* = −0.108, SE = 0.026, 95% CI [−0.161, −0.060]), indicating that a higher growth mindset was associated with a lower external LoC, which in turn reduced AP. In contrast, the indirect effect of internal LoC was not statistically significant (*B* = 0.018, SE = 0.016, 95% CI [−0.012, 0.051]).

**Table 4:** Total, direct, and indirect effects of Growth Mindset on AP through internal and external LoC.

Effect type	Path	B	BootSE	95% CI LL	95% CI UL
Total effect	Growth Mindset **→** Procrastination	−0.170	0.059	−0.288	−0.053
Direct effect	Growth Mindset **→** Procrastination	−0.080	0.059	−0.197	0.037
Total indirect effect	Growth Mindset **→** LoC **→** Procrastination	−0.090	0.027	−0.147	−0.037
Indirect effect	Growth Mindset **→** External LoC **→** Procrastination	−0.108	0.026	−0.161	−0.060
Indirect effect	Growth Mindset **→** Internal LoC **→** Procrastination	0.018	0.016	−0.012	0.051

BootSE, bootstrapped standard error; CI, confidence interval (LL, lower limit; UL, upper limit). The indirect effects were tested using 5,000 bootstrap samples.

Mediation analysis showed that a fixed mindset had a significant positive total effect on AP (*B* = 0.344, SE = 0.057, 95% CI [0.232, 0.456]). Although the direct effect remained statistically significant after the inclusion of mediators (*B* = 0.203, SE = 0.059, 95% CI [0.085, 0.320]), its magnitude decreased (Table 5).

**Table 5:** Total, direct, and indirect effects of fixed mindset on AP through internal and external LoC.

Effect type	Path	B	BootSE	95% CI LL	95% CI UL
Total effect	Fixed Mindset **→** Procrastination	0.344	0.057	0.232	0.456
Direct effect	Fixed Mindset **→** Procrastination	0.203	0.059	0.085	0.320
Total indirect effect	Fixed Mindset **→** LoC **→** Procrastination	0.141	0.028	0.089	0.201
Indirect effect	Fixed Mindset **→** External LoC **→** Procrastination	0.158	0.029	0.102	0.220
Indirect effect	Fixed Mindset **→** Internal LoC **→** Procrastination	−0.016	0.013	−0.045	0.010

BootSE, bootstrapped standard error; CI, confidence interval (LL, lower limit; UL, upper limit). The indirect effects were tested using 5,000 bootstrap samples.

Regarding indirect effects, the total indirect effect of a fixed mindset on AP through internal and external LoC was statistically significant (*B* = 0.141, SE = 0.028, 95% CI [0.089, 0.201]). In particular, external LoC emerged as a significant mediating variable (*B* = 0.158, SE = 0.029, 95% CI [0.102, 0.220]), indicating that a higher fixed mindset was associated with a higher external LoC, which in turn increased AP. In contrast, the indirect effect of internal LoC was not statistically significant (*B* = −0.016, SE = 0.013, 95% CI [−0.045, 0.010]).

The structural relationships tested in this study are visually summarized in Figures 3 and 4. Figure 3 presents the default mediation model for the growth mindset, revealing its direct and indirect effects of growth mindset on AP via internal and external LoC. In contrast, Figure 4 introduces the corresponding structural model for a fixed mindset, showing the direct and indirect pathways linking a fixed mindset to AP through internal and external LoC. These figures provide a visual representation of the standardized path coefficients obtained from the regression and mediation analyses conducted in this study.

**Figure 3:**
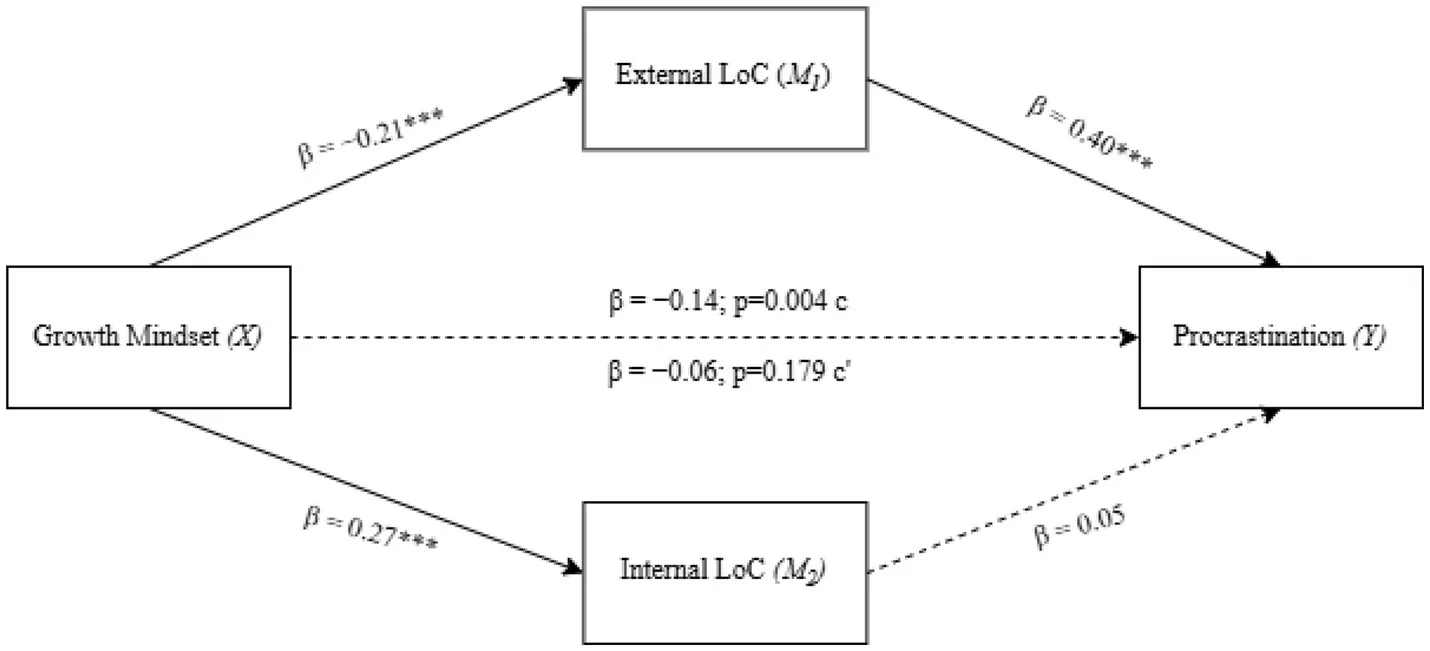
Path diagram of the proposed parallel mediator growth mindset model. Notes: c = total effect; c’ = direct effect; ***p < 0.001.

**Figure 4:**
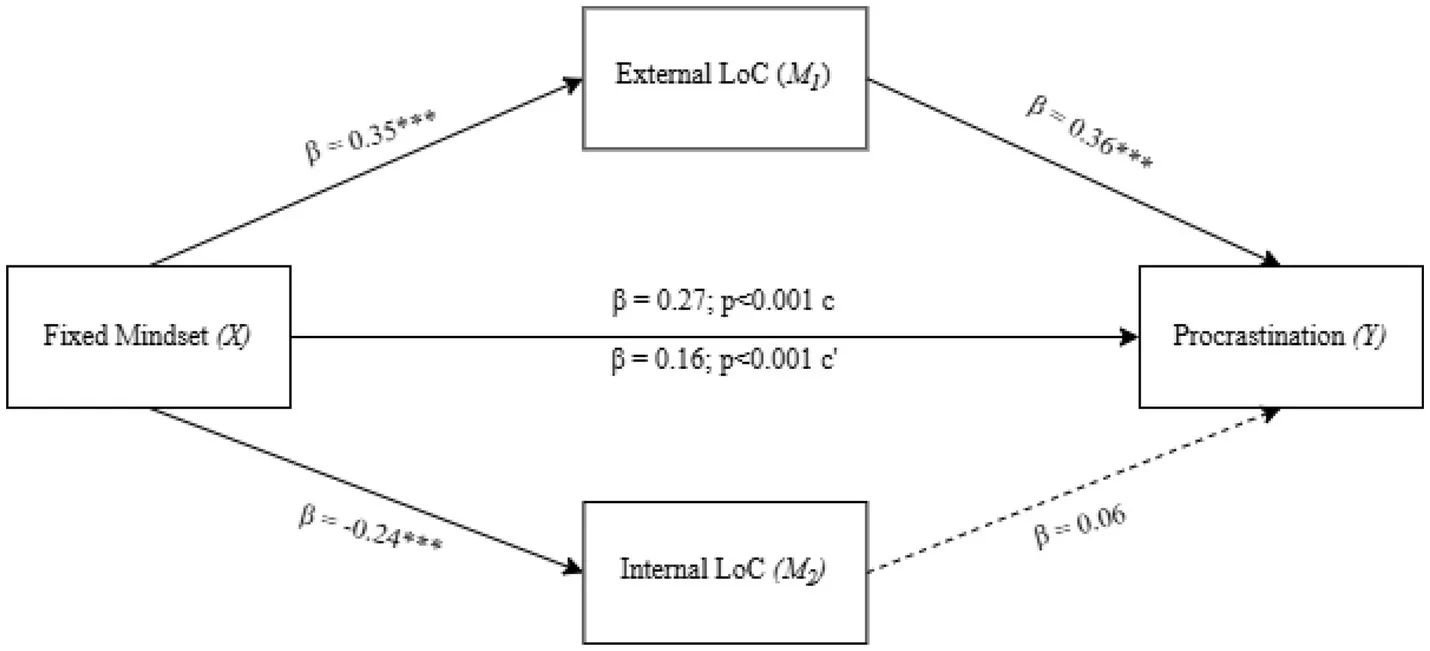
Path diagram of the proposed parallel mediator fixed mindset model. Notes: c = total effect; c’ = direct effect; ***p < 0.001.

## 4 Discussion

The present study examined the direct and indirect relationships between growth mindset, fixed mindset, LoC, and AP among sports science students, focusing on the psychological pathways through which mindset orientations may be associated with procrastination. In this study, internal and external LoC were specifically tested as parallel mediators in the relationship between mindset beliefs and AP. Overall, the findings support the proposed framework by indicating that control-related beliefs, particularly external LoC, may constitute an important pathway linking mindset orientations with AP. Consistent with expectations, a growth mindset showed a positive relationship with internal LoC and a negative relationship with external LoC, whereas a fixed mindset exhibited the opposite pattern. Additionally, external LoC emerged as a significant predictor of AP in both models and served as a key statistical pathway linking mindset beliefs to procrastination. These findings are consistent with mindset theory, which posits that individuals’ beliefs about mindset shape motivational processes (Dweck, 2006).

Bodill and Roberts (2013), who showed that mindset beliefs can shape control orientations, found no significant relationship between a growth mindset and internal LoC in their study of university students, contrary to expectations. Believing that intelligence can be developed did not always lead these students to the belief that “outcomes depend on my effort.” However, the research results confirmed a significant and positive relationship between a fixed mindset and an external academic LoC. In other words, students who believe that intelligence is an innate and unchangeable trait are more inclined to attribute academic outcomes to factors outside their own control (e.g., luck, task difficulty).

In this study, a growth mindset was a negative predictor of AP. However, after internal and external LoC were included in the model, this relationship became non-significant. A notable pattern emerged, indicating that the relationship between growth mindset and AP included a significant indirect association through external LoC. Findings from the growth mindset model showed that students who endorsed stronger development-oriented mindset beliefs reported higher internal LoC and lower external LoC. The mediation findings provided stronger evidence that a growth mindset was indirectly associated with lower levels of AP through external LoC. Specifically, students with stronger growth mindset beliefs were less likely to adopt external LoC beliefs, which were associated with lower levels of procrastination. Interestingly, the indirect effect of internal LoC was not significant, whereas external LoC showed a significant indirect association with AP. One possible explanation is that academic procrastination may be more closely related to perceptions that academic outcomes are determined by external circumstances than to perceptions of personal control over those outcomes. Thus, in the present study, the association between growth mindset and lower AP may have been more closely linked to lower external control beliefs than to stronger internal control beliefs. From a self-regulation perspective, students who attribute academic outcomes to uncontrollable external factors may perceive less personal responsibility for academic tasks, which may be associated with a greater likelihood of delaying task initiation and completion (Bahl et al., 2025; Certel et al., 2017; Özer et al., 2009). In contrast, a growth mindset appears to be associated with lower levels of procrastination primarily through its relationship with maladaptive external attributions. Accordingly, students in sports sciences may not avoid procrastination simply because they strongly believe they can control academic outcomes; rather, they may avoid procrastination when they refrain from attributing outcomes to uncontrollable external factors. In addition, the present findings support previous arguments suggesting that beliefs about intelligence may precede and shape the development of control beliefs (Dweck et al., 1995).

A fixed mindset is a positive predictor of AP, both directly and indirectly. The findings of the fixed mindset model largely overlapped with those obtained in the growth mindset model. A fixed mindset showed a positive relationship with external LoC and a negative relationship with internal LoC. These findings are theoretically meaningful because students who believe that their intelligence and abilities are fixed may perceive their opportunities to influence academic outcomes through effort as limited. Consequently, they may be more likely to attribute success and failure to external factors rather than personal actions. Such a belief system may be associated with a diminished sense of control over academic outcomes and greater vulnerability to procrastination (Mataracı and Alemdağ, 2024; Steel, 2007). Consistent with this interpretation, a fixed mindset showed a significant direct effect on AP and a significant indirect effect through external LoC. Similar to the growth mindset model, the indirect path via the internal LoC was not statistically significant. Notably, internal LoC did not play a significant mediating role in either model. This suggests that AP behavior may be more strongly associated not merely with high individual perceptions of control, but specifically with the predominance of external control beliefs. Considering that the effect of a growth mindset is context-sensitive (Lou and Li, 2023; Macnamara and Burgoyne, 2023), societal norms and unique educational environments, such as sports sciences as a specific academic field, may either strengthen or weaken the effect of a growth mindset.

Bahl et al. (2025) findings indicate that academic performance is related not only to cognitive competencies but also to students’ perceptions of control over academic outcomes and the self-regulation processes shaped by these perceptions. In particular, findings suggesting that an external LoC may negatively affect academic outcomes by increasing AP behavior highlight the importance of students developing a sense of personal control and responsibility for their learning processes.

The present findings can also be considered within the broader contemporary literature on AP. Recent evidence indicates that AP is associated with multiple psychological, self-regulatory, and technology-related factors (Özbay et al., 2025). Building upon these empirical perspectives, academic procrastination operates within a broader ecosystem of psychological and behavioral processes (Karagöz and Çınar Özbay, 2025). Within this framework, the prominence of external locus of control clarifies how cognitive mindsets translate into task-avoidance behaviors under varying demands. Taken together, the present study extends this line of inquiry by highlighting mindset orientations and LoC as distinct psychological pathways associated with AP.

Although sport-specific contextual factors were not directly measured in the present study, sports science students often encounter unique structural demands related to training, competition, travel, and physical performance alongside their academic responsibilities (De Maio et al., 2025; Mabala et al., 2026). In such environments, academic stressors are frequently regulated externally (Condello et al., 2019; Mabala et al., 2026). Consequently, under strong external or structural pressures, the protective influence of an internal LoC may be less pronounced (De Maio et al., 2025). If the controllability of such external constraints is perceived as low, students may become more inclined to attribute their academic outcomes to environmental conditions or time pressure. This tentative contextual consideration may help explain why external LoC emerged as a prominent indirect pathway in our findings, whereas internal LoC did not. However, future empirical research directly assessing sport-specific loads is needed to confirm these potential contextual nuances.

In addition to the default relationships, the control variables also revealed noteworthy findings. In particular, even after accounting for mindset beliefs and LoC, gender and age remained significant AP predictors. Gender emerged as a significant predictor associated with AP in both models, indicating that male students reported higher AP levels than female students. This finding is consistent with prior research suggesting that male students tend to engage in higher levels of procrastination due to differences in self-regulation, time management skills and academic responsibility (Certel et al., 2017; Özer et al., 2009). Although the present study did not specifically investigate the underlying mechanisms of these gender differences, the findings indicate that gender continues to be an important factor associated with procrastination among sports science students. Age was negatively correlated with AP in both models, indicating that older students were less prone to procrastination than their younger peers. One potential explanation is that enhanced academic experience and maturity may be associated with more effective self-regulation and time-management skills. As students progress through their university education, they may become better equipped to cope with academic demands and deadlines, thus reducing their tendency to procrastinate on academic tasks. Certel et al. (2017) reported that male athletes had significantly higher external academic LoC and AP scores than female athletes. In a previous study conducted in the general population, procrastination was more prevalent in the youngest age group, and in that group, males procrastinated more than females (Beutel et al., 2016). Steel (2007) also reported that procrastination behavior decreases with age and is higher among males than females. The findings of these studies support the results of the present study.

## 5 Conclusion

This study concludes that, among sports science students, academic procrastination was associated with students’ perceptions of control over academic outcomes. Our parallel mediation models indicated that external locus of control was a significant statistical pathway linking mindset orientations to academic procrastination, whereas internal locus of control did not significantly mediate these relationships. A fixed mindset was associated with greater reliance on external control beliefs and higher levels of academic procrastination, whereas a growth mindset was associated with lower external control beliefs and lower academic procrastination. These findings suggest that educational interventions addressing academic procrastination may benefit from considering both students’ beliefs about the development of abilities and their perceptions of control. However, given the cross-sectional design, these relationships should be interpreted as associations rather than causal effects.

## 6 Limitations and recommendations

This study has certain limitations. As this study was conducted using a cross-sectional design, the relationships between variables should not be interpreted causally. In future studies, the temporal relationships among mindset beliefs, LoC, and AP can be examined in more detail by employing longitudinal or experimental designs. In this study, data were collected at a single time point using self-report instruments, which may increase the possibility of common method bias. Future research should aim to provide a more comprehensive explanation of the psychological mechanisms of AP by examining the motivational processes, such as achievement goals, self-regulation, and academic self-efficacy, that underlie the relationship between mindset and LoC. Finally, the data were collected only from enrolled sports science students at two universities located in Eastern Anatolia, which may limit the generalizability of the findings to other geographical regions or various academic disciplines. The study also did not include a comparison group from other university disciplines or assess athletic status, training load, or other sport-specific contextual factors. Therefore, the present findings do not allow us to determine whether the observed relationships differ from those among students in other academic disciplines or according to sport-related characteristics. Future studies should address these limitations by incorporating comparison groups and examining sport-specific factors as potential contextual or moderating variables. Such research could also cross-validate the parallel mediation models using multicenter or cross-cultural samples.

## Data Availability

The raw data supporting the conclusions of this article will be made available by the authors, without undue reservation.
